# Lewis^y^ Promotes Migration of Oral Cancer Cells by Glycosylation of Epidermal Growth Factor Receptor

**DOI:** 10.1371/journal.pone.0120162

**Published:** 2015-03-23

**Authors:** Wei-Ling Lin, Yi-Shiuan Lin, Guey-Yueh Shi, Chuan-Fa Chang, Hua-Lin Wu

**Affiliations:** 1 Department of Biochemistry and Molecular Biology, National Cheng Kung University, Tainan, Taiwan; 2 Cardiovascular Research Center, National Cheng Kung University, Tainan, Taiwan; 3 Department of Medical Laboratory Science and Biotechnology, National Cheng Kung University, Tainan, Taiwan; University of Central Florida, UNITED STATES

## Abstract

Aberrant glycosylation changes normal cellular functions and represents a specific hallmark of cancer. Lewis^y^ (Le^y^) carbohydrate upregulation has been reported in a variety of cancers, including oral squamous cell carcinoma (OSCC). A high level of Le^y^ expression is related to poor prognosis of patients with oral cancer. However, it is unclear how Le^y^ mediates oral cancer progression. In this study, the role of Le^y^ in OSCC was explored. Our data showed that Le^y^ was upregulated in HSC-3 and OC-2 OSCC cell lines. Particularly, glycosylation of epidermal growth factor receptor (EGFR) with Le^y^ was found in OC-2 cells, and this modification was absent upon inhibition of Le^y^ synthesis. The absence of Le^y^ glycosylation of EGFR weakened phosphorylation of AKT and ERK in response to epidermal growth factor (EGF). Additionally, EGF-triggered cell migration was reduced, but cell proliferation was not affected. Le^y^ modification stabilized EGFR upon ligand activation. Conversely, absence of Le^y^ glycosylation accelerated EGFR degradation. In summary, these results indicate that increased expression of Le^y^ in OSCC cells is able to promote cell migration by modifying EGFR which in turn stabilizes EGFR expression and downstream signaling. Targeting Le^y^ on EGFR could have a potential therapeutic effect on oral cancer.

## Introduction

Oral cancer is a type of head and neck cancer that can arise from any part of the oral cavity. Tobacco [1] and alcohol [2] use are the major risk factors of oral cancer, which is a leading cause of death among middle-aged people. Oral cancer may be curable with early diagnosis and treatment; however, the survival rate is decreased in the advanced-stages. [3] Depending on the originating tissues, there are several types of oral cancers. Oral squamous cell carcinoma (OSCC), which occurs in the lining mucosa of the mouth and lips, is the most common and has become an epidemiological issue around the world. [4]

Tumor progression is often associated with atypical glycosylation of cell surface proteins. [5] Lewis^y^ (Le^y^, Fucα1-2Galβ1-4(Fucα1-3)GlcNAcβ1-R) is a difucosylated oligosaccharide that has been found to be overexpressed in a variety of tumors, especially in epithelium-derived cancers. [6] Also, Le^y^ expression is associated with clinical stage and progression of tumors. [7,8] The upregulation of Le^y^ in tumors promotes cell adhesion [9,10], proliferation [11,12], migration [13], and resistance to chemotherapy. [13–15] Inhibition of Le^y^ with antibodies [13,16] or suppression of Le^y^ synthesis can effectively inhibit growth and migration of tumor cells. [17] The abundant expression of Le^y^ precursor in the outgrowth of the epithelium was found in human oral mucosa with a three-day wound [18], where the increased expression of Le^y^ precursor is related to increased cell motility. In addition, increased Le^y^ expression is significantly related to poor prognosis in oral cancer patients. [19] However, the function of Le^y^ in oral cancer is not completely understood.

Epidermal growth factor receptor (EGFR), a member of the ErbB family, regulates many cellular functions through activation and phosphorylation of its intrinsic tyrosine kinase domain and downstream signaling molecules. [20] EGFR overexpression has been observed in oral cancer [21], where it may promote cancer progression. Therefore, EGFR is proposed to be a target for anticancer therapy in oral cancer. [22] Furthermore, glycosylation of EGFR is essential for its normal functions, such as ligand-binding, dimerization, signal transduction, and the internalization-recycling pathway. [23] Le^y^ glycosylation of EGFR has been found to modulate the function of EGFR. [12,16,24] However, whether the expression of Le^y^ in oral cancer would regulate EGFR’s functions by glycosylation is unknown. Thus, the aim of the study was to investigate the functional role of Le^y^ and the association of Le^y^ with EGFR in oral cancer. Herein, we demonstrate that Le^y^ modification of EGFR stabilizes EGFR expression and downstream signaling and promotes migration in OSCC cells. Our results provide insight into how Le^y^ glycosylation manipulates EGFR signaling in oral cancer and indicate that Le^y^ is a potentially important biomarker and treatment target in Le^y^-overexpressing oral cancers.

## Materials and Methods

### Cell Culture

Three human OSCC cell lines, OC-2, OEC-M1, and HSC-3, and one immortalized human gingiva keratinocytes, SG, were used in the present study. All of these cells were kindly provided by Professor Dar-Bin Shieh and Yuh-Ling Chen (National Cheng Kung University). OC-2, which are derived from a primary tumor of the buccal mucosa of a Chinese man with a history of smoking and betel-nut chewing [25], and OEC-M1, which are derived from a primary tumor of the gingiva of an adult male OSCC patient from Taiwan with a history of betel-quid chewing [26], were cultured in RPMI1640. HSC-3, which are derived from human tongue carcinoma with lymph node metastasis [27], was cultured in MEM. SG, an immortalized human gingival epithelial cell line derived from clinically normal adult human gingiva, was cultured in DMEM. All of the culture media (GIBCO BRL, Grand Island, NY, USA) were supplemented with 10% (v/v) fetal bovine serum (FBS; Sigma-Aldrich, St. Louis, MO, USA), 2 mmol/L L-glutamine (Sigma-Aldrich, St. Louis, MO, USA), 500 U/mL penicillin and 100 μg/mL streptomycin (Sigma-Aldrich, St. Louis, MO, USA). Cells were cultured in a humidified incubator at 37°C with 5% CO_2_.

### Western Blotting

Cells were washed with ice-cold phosphate-buffered saline (PBS) three times and treated with cell lysis buffer (Cell Signaling Technology, Danvers, MA, USA). Total protein concentration of cell lysate was quantified using a bicinchoninic acid protein assay. Samples were equally loaded and subjected to electrophoresis. After electrophoresis, the SDS-PAGE gel was blotted on to a nitrocellulose membrane (Merck Millipore, Darmstadt, Germany) and incubated with primary antibodies, which were detected using peroxidase-conjugated secondary antibodies. Signals were visualized using enhanced chemiluminescence.

### Quantitative Real-Time PCR

Total cell RNA was extracted using a Total RNA mini extraction kit (RBC Bioscience, Taiwan) according to the manufacturer’s instructions. Two micrograms of isolated RNA was reverse-transcribed to cDNA. The reactions for real-time PCR were performed using an ABI 7500 sequence detection system (Life Technologies, Grand Island, NY, USA), as described previously. [28] The relative mRNA expression level was calculated by using 2^-ΔCT^ method. [29] Glyceraldehyde 3-phosphate dehydrogenase (*GAPDH*) was used as the reference gene. Primers sequences are listed in Table 1.

**Table 1:** Primers used in quantitative real-time PCR.

**Gene**	**Primer sequence**
**GAPDH**	Fwd: aggtcatccctgagctgaacgg
	Rev: cgcctgcttcaccaccttcttg
**FUT1**	Fwd: gcaggccatggactggtt
	Rev: cctgggaggtgtcgatgttt
**FUT2**	Fwd: ctcgctacagctccctcatctt
	Rev: cgtgggaggtgtcaatgttct
**FUT4**	Fwd: gagctacgctgtccacatcacc
	Rev: cagctggccaagttccgtatg

### Lentiviral Delivery of Short Hairpin RNA

All lentiviral vectors and the viral delivery system were established by and obtained from the National RNAi Core Facility (Academia Sinica, Taipei, Taiwan). In brief, recombinant lentiviruses were produced by cotransfection of HEK293T cells with pMD.G, pCMVΔR8.91, and pLKO.1-puro vectors containing respective short hairpin RNAs. To knockdown of fucosyltransferase 1 (FUT1) expression, FUT1-specific short hairpin RNA (shFUT1) with the targeting sequence of 5′-ACTTGAGAGATCCTTT-3′ was used. Luciferase-specific short hairpin RNA (shLuc) with the targeting sequence of 5′-TCACAGAATCGTCGTATGCAG-3′ was used as a negative control. The recombinant virus containing media were harvested 24- and 48-h post transfection, and the viral load was titered by infecting A549 cells with serial dilutions. For infection, OC-2 cells were seeded and grown for 24 h, followed by replacement of the culture medium with the recombinant virus containing medium (multiplicity of infection of 10) and culturing overnight. Infected cells were selected using puromycin (2 μg/mL) and prepared for further experiments.

### Immunoprecipitation

Total cell lysates (500 μg) were incubated with primary antibodies (1 μg) at 4°C for 8–12 h, followed by precipitation with Protein-G agarose beads (Sigma-Aldrich, St. Louis, MO, USA) at 4°C for 4 h. After washing, the precipitates were separated by SDS-PAGE and transferred onto nitrocellulose membranes. The membranes were blocked using 5% milk in PBS/0.01% Tween 20 and blotted with the relevant antibodies.

### Assay of AKT and ERK Phosphorylation

Cells were starved for 24 h and treated with EGF (ProSpec, Ness-Ziona, Israel) under the indicated conditions. Cell lysates were prepared for Western blotting for phosphorylated AKT (clone sc-7985-R; Santa Cruz Biotechnology, Santa Cruz, CA, USA) or phosphorylated ERK (clone sc-7383; Santa Cruz Biotechnology, Santa Cruz, CA, USA).

### Cell Proliferation Assay

Cells (3 × 10^3^/well) were seeded in medium containing 2% FBS onto a 96-well plate and grown for 24 h. Then, cells were treated with EGF as indicated at 37°C, followed by incubation with WST-1 reagent (Roche, Indianapolis, IN, USA). After 2 h of incubation, cell proliferation was determined by measuring the absorbance at 450 nm.

### Scratch Wound Healing Assay

Cells (2 × 10^6^/well) were seeded onto a 6-well plate and grown to confluence in medium containing 10% FBS. A scratch was introduced to the confluent cell sheet with a 200-μL pipette tip. After washing with PBS, cells were treated with EGF under the indicated conditions, and allowed to migrate for 10 h. The migration of cells was recorded every 30 min by time-lapse microscopy.

### Preparation of Cell Membrane Proteins

EGF-treated cells were harvested in homogenization buffer [20 mM Tris-HCl (pH 7.5), 2 mM EDTA (pH 8.0), 5 mM EGTA (pH 8.0), 10% glycerol, and protease inhibitor cocktail (Sigma-Aldrich, St. Louis, MO, USA)] and lysed on ice using an ultrasonic homogenizer. Cellular debris was removed by centrifugation (2,500 × *g*, 10 min). The supernatant was collected and centrifuged at 15,000 × *g* for 30 min. The pellet was collected and suspended in 100 μL of homogenization buffer containing 1% NP-40. The protein concentration was quantified using bicinchoninic acid protein assay.

### EGF Binding Assay

Cells were incubated with serum-free medium containing Alexa Fluor 488 conjugated EGF (Molecular Probes, Grand Island, NY, USA) for 1 h at 4°C to prevent EGF/EGFR complex internalization. After washing with ice-cold PBS, cells were harvested in cell dissociation buffer (Sigma-Aldrich, St. Louis, MO, USA) by gently scraping on ice. Cells were then fixed in 4% formaldehyde for 15 min, and the surface bound Alexa Fluor 488 conjugated EGF was analyzed using FACS Calibur (BD Biosciences, Franklin Lakes, NJ, USA). Fluorescence intensity was analyzed using FlowJo software (FlowJo LLC, Inc., Ashland, OR, USA). To determine EGF binding specificity, a 10× concentration of unlabeled EGF was added to compete with labeled EGF.

### EGFR Dimerization Assay

Cells were starved with serum-free medium for 24 h and treated with EGF for 1.5 min. After washing with ice-cold PBS, 3 mL of BS3 compound (Thermo Fisher Scientific, Waltham, MA, USA) were added and incubated with cells on ice for 2 h to cross-link with EGFR. The reaction was quenched at RT for 15 min by treatment with Tris-HCl (50 mM, pH 7.5). After washing with ice-cold PBS, cells were harvested and lysed for western blotting for EGFR. The dimerized EGFR showed an approximate molecular mass of 340 kDa.

### Statistics

Data are represented as mean ± SEM. Statistical significance between two groups was analyzed using an unpaired Student *t*-test. Comparison of group of three or more were done using one-way ANOVA with a post test of Tukey correction. A P-value < 0.05 was considered statistically significant.

## Results

### Le^y^ Expression in Human OSCC Cell Lines

**Fig 1:**
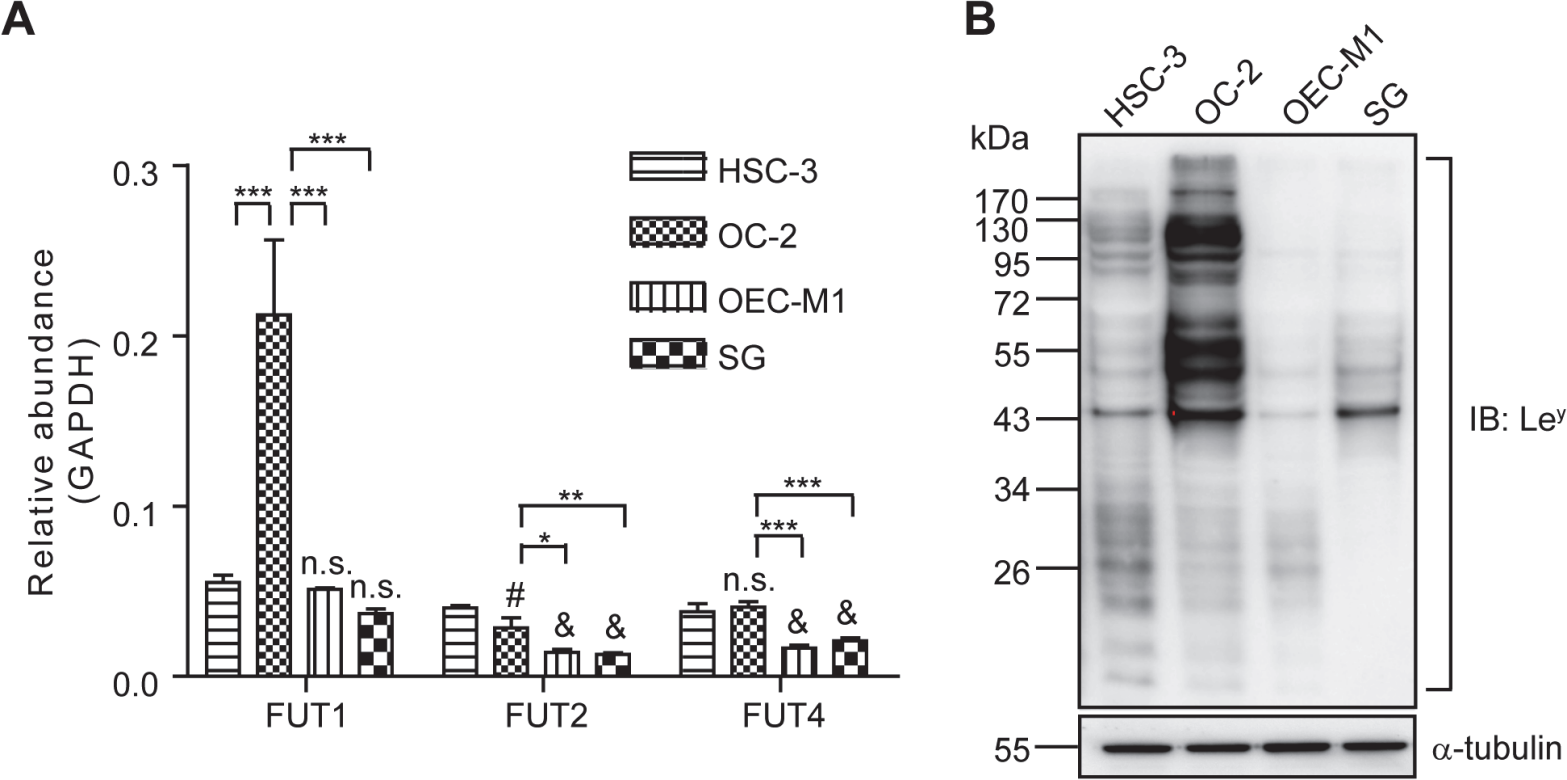
Expression of Le^y^ in Oral Cells. (**A**) Analysis of FUT1, FUT2, and FUT4 mRNA expression in four oral cell lines as determined using real-time PCR. The relative abundance of transcripts was obtained by normalization to GAPDH. Data represent the mean ± SEM (n = 3). The relative abundance shown in the y-axis was 100 times the results. n.s.: not significant, #*P* < 0.05 and &*P* < 0.001 compared to HSC-3. *P < 0.05, *P < 0.01 and ***P < 0.001. (**B**) Representative Western blot of Le^y^ and α-tubulin in four oral cell lines.

We determined the expression of Le^y^ synthesis enzymes, FUT1, 2, and 4 [30], in three human OSCC cell lines, HSC-3, OC-2, and OEC-M1, and one immortalized human gingival keratinocytes, SG, using real-time PCR. The results showed that the mRNA level of FUTs was higher in HSC-3 and OC-2 than in OEC-M1 and SG cells (Fig. 1A). Additionally, Le^y^ expression was evaluated by Western blotting, and the most abundant expression level of Le^y^ was observed in OC-2 cell line (Fig. 1B).

### Le^y^ is Glycosylated of EGFR in OC-2 Cells

**Fig 2:**
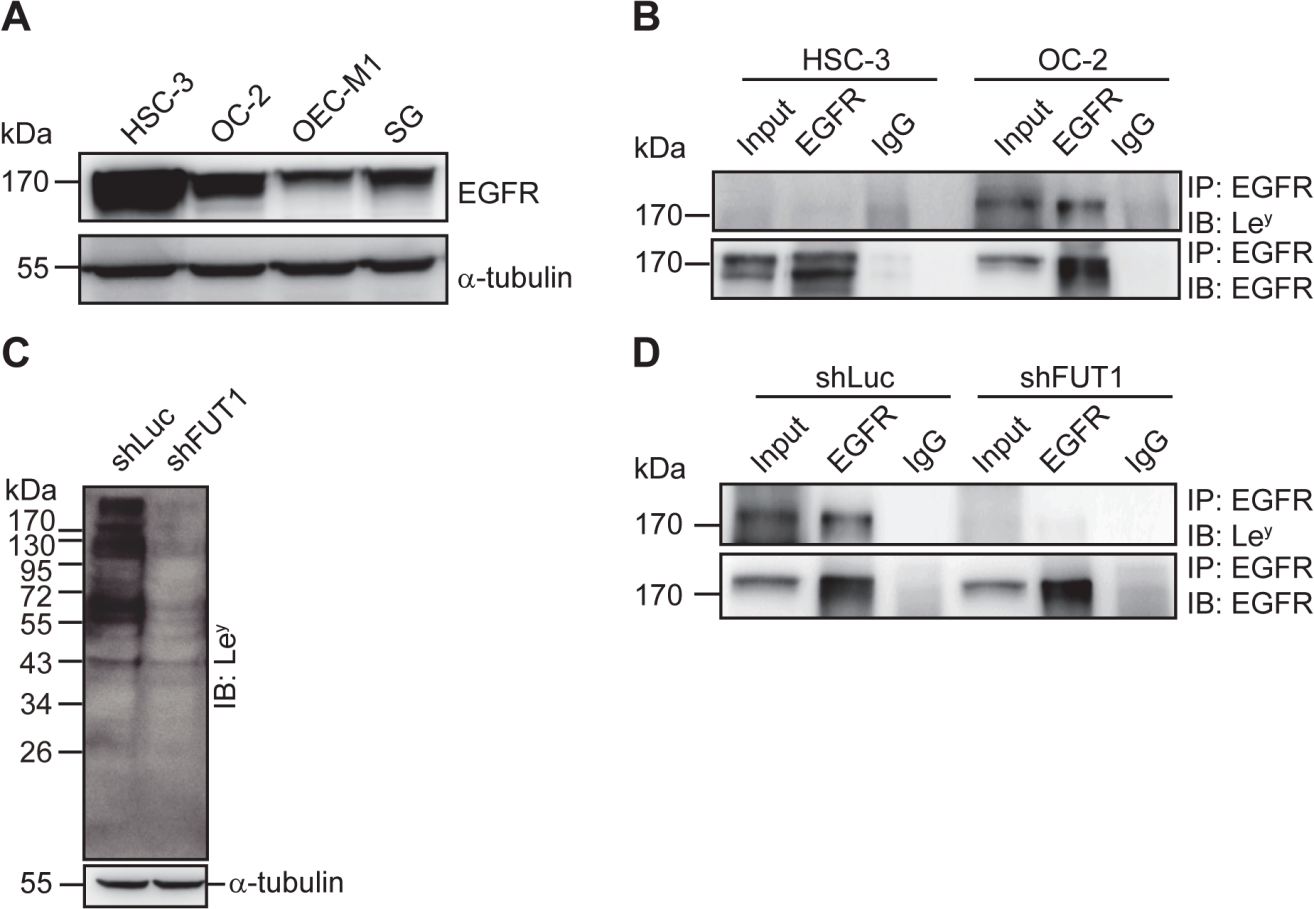
Le^y^ Glycosylation of EGFR in OC-2 Cells. (**A**) Representative Western blot of EGFR and α-tubulin in four oral cell lines. (**B**) Total cell lysates were used for immunoprecipitation (IP) with anti-EGFR and isotype control antibodies and immunoblotted (IB) using anti-Le^y^ and anti-EGFR antibodies. (**C**) Representative Western blot of Le^y^ and α-tubulin in OC-2 cells transduced with FUT1 (shFUT1) and control shRNA (shLuc). (**D**) Total cell lysates prepared from OC-2 cells with FUT1 (shFUT1) and control shRNA (shLuc) transduction were used for immunoprecipitation (IP) with anti-EGFR and isotype control antibodies, followed by immunoblotting (IB) with anti-Le^y^ and anti-EGFR antibodies.

Overexpression of EGFR in OSCC patients has been reported. [21] Similarly, in the present study, we found that the OSCC cell lines, HSC-3 and OC-2, showed higher EGFR expression than the other two oral cell lines (Fig. 2A). To further examine whether Le^y^ could be glycosylated of EGFR, we performed immunoprecipitation in HSC-3 and OC-2 cells. As shown in Fig. 2B, Le^y^ was expressed on EGFR in OC-2 cells, but not in HSC-3. We suppressed Le^y^ expression using shRNA depletion of FUT1, which is the key enzyme for Le^y^ synthesis in OC-2 cells. After transduction with lentiviral shRNA vectors, the expression level of Le^y^ and the modification of EGFR were successfully repressed. There were no changes in Le^y^ and the modification of EGFR in control (shLuc) transduced cells (Fig. 2C, D).

### EGF-induced Phosphorylation and Migration is Attenuated upon Suppression of Le^y^ Expression

**Fig 3:**
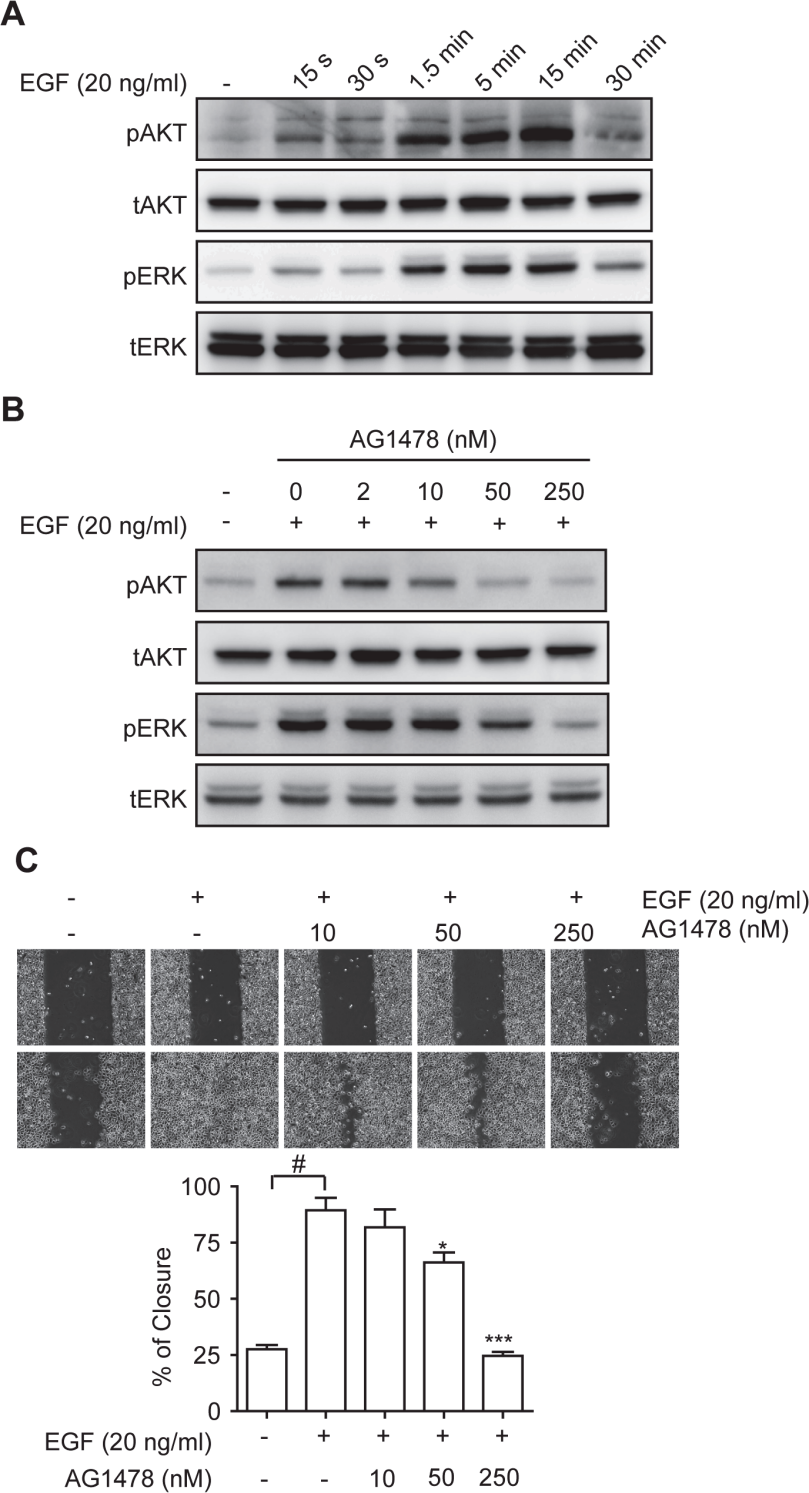
Effects of EGF Treatment in OC-2 Cells. (**A**) Cells were stimulated with EGF for the indicated duration, and total cell lysates were used for Western blotting using the indicated antibodies. (**B**) Cells were stimulated with (+) or without (-) EGF for 5 min in the presence of various doses of AG1478 (EGFR inhibitor), and total cell lysates were used for Western blotting using the indicated antibodies. (**C**) After preincubation of various dosages of AG1478 (EGFR inhibitor) for 30 min, cells were wounded and stimulated with EGF. The progress of cell migration was recorded by using time-lapse microscopy, and the percentage of wound closure was calculated. Representative images demonstrating wound closure with EGF and EGFR inhibitor treatment as indicated. Quantitative data are shown. Data are presented as the means ±SEM (n = 3). *P < 0.05 and ***P < 0.001 versus EGF only. #*P* < 0.001.

To characterize the effects of EGFR activation in OC-2 cells, we stimulated cells with EGF and analyzed the phosphorylation of downstream signal molecules AKT and ERK. The data showed that AKT and ERK were phosphorylated by EGF stimulation in a time-dependent manner (Fig. 3A), whereas, pretreatment with the tyrosine kinase inhibitor, AG1478, dose-dependently reduced the signal transduction (Fig. 3B). Next, we performed proliferation and migration assays to examine the functions of EGFR in OC-2 cells. The results showed that there were no obvious differences in the proliferation of EGF-treated OC-2 cells compared with that in the control group (S1A Fig.). Similarly, pretreatment with AG1478 had no significant effect on cell proliferation (S1B Fig.). In contrast, cell migration was enhanced by EGF stimulation, which was inhibited by AG1478 (Fig. 3C). These observations indicate that overexpression of EGFR in OC-2 cells can increase cell migration.

**Fig 4:**
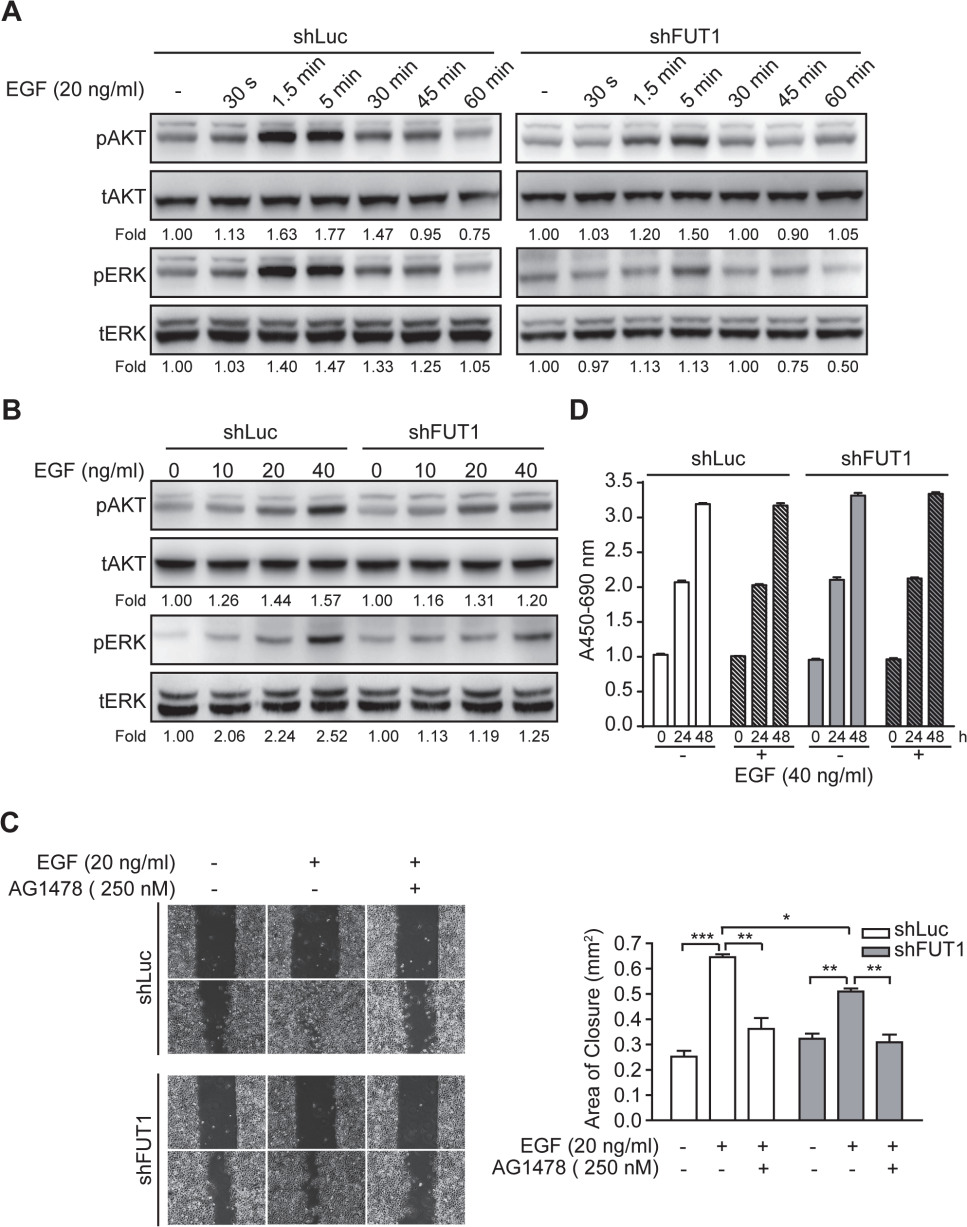
Le^y^ Suppression Attenuates EGF-induced Phosphorylation and Migration in OC-2 Cells. Cells were stimulated with EGF for the indicated durations (**A**) or with various doses of EGF for 1.5 min (**B**). Total cell lysates were used for Western blotting using the indicated antibodies. Western blots from three independent experiments were analyzed by densitometry. The indicated fold changes represent the density relative to control (-). (**C**) After preincubation of AG1478 (EGFR inhibitor) for 30 min, each cells was wounded and stimulated with EGF. The progress of cell migration was recorded by using time-lapse microscopy, and the area of wound closure was calculated. Representative images demonstrating wound closure with EGF and EGFR inhibitor treatment as indicated. Quantitative data are shown. Data are presented as the means ± SEM (n = 3). *P < 0.05, **P < 0.01, and ***P < 0.001. (**D**) OC-2 cells transduced with shLuc or shFUT1 were stimulated with EGF in the presence or absence of AG1478 (EGFR inhibitor), and cell growth was analyzed every 24 h using WST-1 reagent. Data represent the mean ± SEM (n = 3).

Glycosylation leads to conformational alteration, which is critical for the activity of EGFR. [23] We determined the effects of Le^y^ modification on the function of EGFR in OC-2 cells by treating control and FUT1 knockdown OC-2 cells with serial times or multiple doses of EGF, and analyzing the phosphorylation of AKT and ERK. The data showed that 5 min of EGF stimulation in control cells resulted in ∼80% and ∼50% increases in pAKT and pERK levels, respectively. The same treatment resulted in 50% and 10% increases in pAKT and pERK levels in FUT1 knockdown cells, respectively (Fig. 4A). Similarly, the average fold changes of pAKT and pERK levels were lower in knockdown cells than in control (∼20% versus ∼60% increase in pAKT and ∼30% versus ∼150% increase in pERK) at the ultimate dose of EGF (Fig. 4B). Moreover, EGF-mediated cell migration was decreased in Le^y^-deficient cells (Fig. 4C), whereas cell proliferation showed no significant difference when compared with that in control cells (Fig. 4D). Therefore, Le^y^ may be involved in EGFR-mediated signal transduction and cell migration.

### Le^y^ Glycosylation Stabilizes EGFR Expression, but Has No Effects on Ligand Binding and EGFR Dimerization

**Fig 5:**
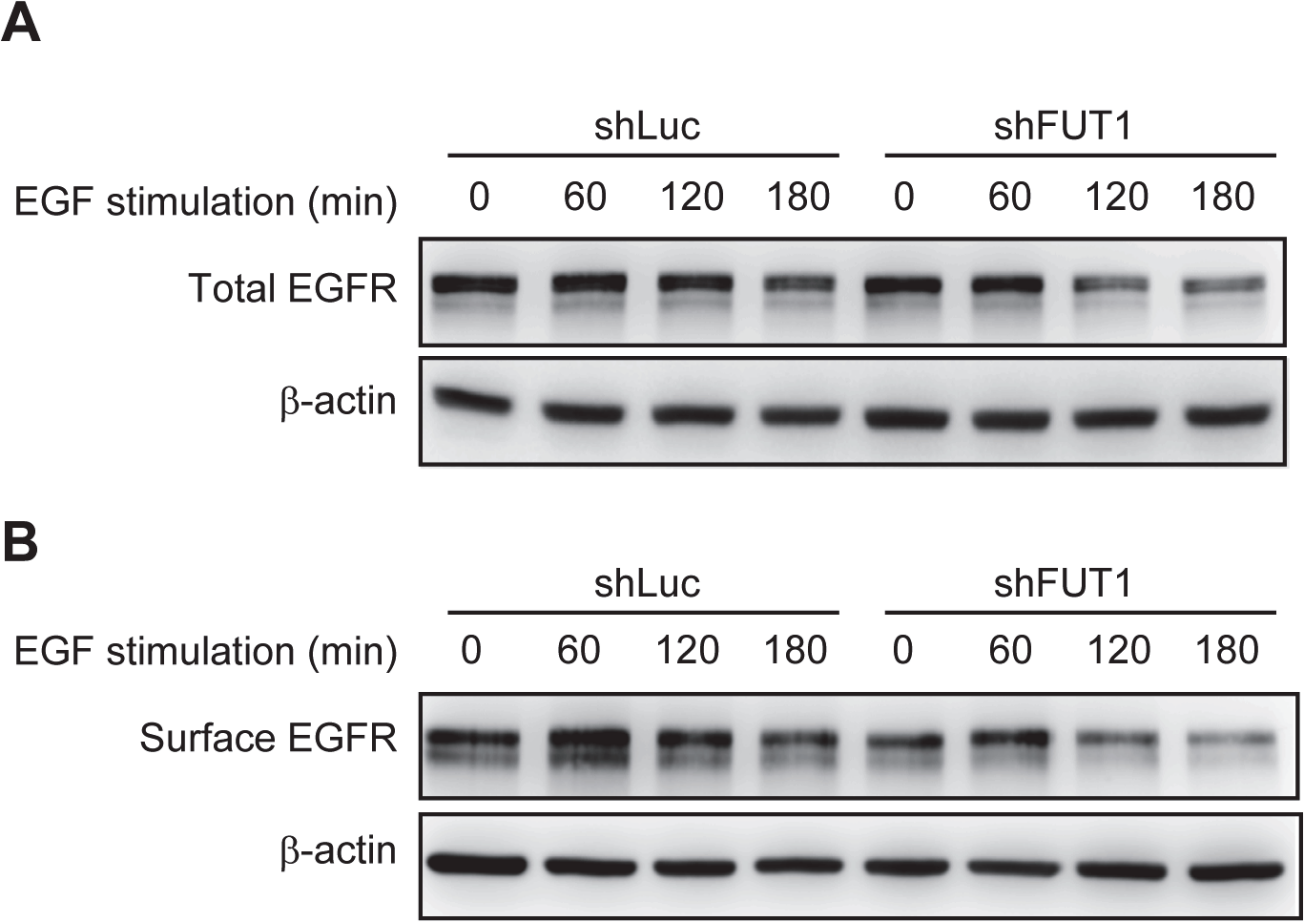
Le^y^ Suppression Accelerates Ligand-Induced Degradation of EGFR. After 24 h of serum-starvation, cells were treated with 40 ng/mL EGF for the indicated durations, and total cell lysates (**A**) or cell membrane fractions (**B**) were used for Western blotting using anti-EGFR antibody.

To further investigate how Le^y^ modification influenced the activity of EGFR, ligand binding and dimerization of EGFR upon activation were tested. Neither events was altered by FUT1 knockdown (S2 Fig.). After EGF-EGFR binding, the complex undergoes internalization and degradation. [31] This process leads to clearance of the activated receptors from the cell surface, thereby terminating signal transduction. Thus, we next questioned whether knockdown of FUT1 would affect degradation of the complex. After EGF treatment, EGFR was stably expressed for 120 min in control cells. In cells transduced with shFUT1, the levels of total and surface EGFR were significantly decreased at the same time point (Fig. 5). This suggests that the expression of Le^y^ in OC-2 cells modified EGFR to maintain its activity, leading to enhanced cell migration.

## Discussion

Elevated Le^y^ expression has been reported in many types of tumors, and it has been associated with the aggressiveness of the tumor cells. In the current study, we demonstrated that Le^y^ is highly expressed in HSC-3 and OC-2 OSCC cell lines (Fig. 1). Le^y^ modification is carried by EGFR in OC-2 cells (Fig. 2B). To explore the role of Le^y^ glycosylation in EGFR-mediated functions and the correlation with tumor malignancy, OC-2 cells with FUT1 knockdown were produced for assays of signal transduction, cell proliferation, and migration. Our results showed that EGFR activation promotes cell migration, but not proliferation, in OC-2 cells (Fig. 3), in which Le^y^ is necessary for maintenance of EGFR functions. Loss of Le^y^ attenuates phosphorylation of AKT and ERK in the downstream EGFR signaling pathway and results in the reduction of cell migration (Fig. 4). We further elucidated that Le^y^ can stabilize EGFR expression (Fig. 5). This finding implies that overexpression of Le^y^ is associated with malignancy by modifying EGFR, thereby enhancing cancer cell migration.

Overexpression of EGFR is detected in various tumors and augmented activation of EGFR triggers multiple cellular effects that promote tumor progression. [32] Le^y^-regulated EGFR signaling pathway has been demonstrated in other types of cancer, including ovarian cancer [12], breast cancer [16,24], and epidermoid carcinoma. By manipulation of the expression and activity of Le^y^, these studies described that the modification of Le^y^ in EGFR enhances the activation of downstream signals to mediate cell proliferation. In the current study, we found that Le^y^ was glycosylated of EGFR in OC-2 cells and it modulated AKT and ERK signaling pathways to facilitate cell migration. This finding provides another evidence supporting the pro-tumoral role of Le^y^. Notably, Le^y^ may not be able to control the growth of OC-2 cells via regulating EGFR, since we found that the proliferation of OC-2 cells was not altered in response to EGF or AG1478 treatment. Earlier studies revealed that the endogenous phosphorylation of aurora kinases, which are mitosis regulators, is detected in OC-2 cells. [33] This suggests that OC-2 cells are capable of increasing cell-cycle progression in the absence of additional growth factor treatment, and which may explain why EGF did not trigger higher cell proliferation in comparison to that with control treatment.

Upon EGF binding to EGFR, the endocytic pathway is activated to downregulate EGFR expression through degradation or recycling. [31] Antibodies direct against Le^y^ have been characterized to have inhibitory effects on ErbB1 (EGFR) and ErbB2 signaling pathways through affecting the intracellular routing of the ErbB receptors, but not by abrogating EGF binding to receptors. [16] Here, we demonstrated that the amount of EGFR expression in response to EGF was reduced in the absence of Le^y^ modification. These observations indicate that Le^y^ structure on EGFR essentially maintains the receptor stability upon activation, and whether Le^y^ would exhibit the similar function on other kinase receptors should be further investigated.

HSC-3 cells are derived from metastatic tongue cancer tissues and are considered highly invasive. [27] However, despite the proposed theory that Le^y^ would correlate positively with the malignancy of oral cancer, HSC-3 expressed less Le^y^ than OC-2 cells, and EGFR was not modified with Le^y^ in these cells. Additionally, the levels of FUT2 were higher in HSC-3 than in other oral cells, while OC-2 expressed the most FUT1 of all the cell lines tested. The variance of Le^y^ and FUT expression observed here indicates that there are distinguished glycosylation profiles among types of OSCC. This observation requires further confirmation by large-scale analysis. As mentioned earlier, Le^y^ significantly correlates with poor prognosis in oral cancer patients. Here, we further identified that Le^y^ expression is cell type-dependent. Thus, Le^y^ may serve as a glycan marker utilizing for diagnosis and treatment.

Anti-EGFR therapy is becoming a standard strategy for cancer therapy; however, there are some side effects caused by anti-EGFR agents because of damage to EGFR expressing normal tissues. [34] Conversely, Le^y^ is frequently overexpressed on epithelium-derived cancers [6] with limited or no expression in adult healthy tissues. [35] Therefore, Le^y^ may be an alternative target to abolish EGFR function in tumor tissues specifically. Most importantly, no significant side effects were observed in trials of anti-Le^y^ antibody treatment. [36]

Taken together, these studies emphasize that oral cancer cells with Le^y^ overexpression exhibit enhanced cell mobility. The role of Le^y^ is to stabilize EGFR and maintain EGFR-triggered signaling pathways. Our results also provide insight into how Le^y^ glycosylation manipulates EGFR signaling in oral cancer. Accordingly, Le^y^ may become a biomarker or treatment target in Le^y^-overexpressing oral cancers.

## Supporting Information

S1 Fig EGF Treatment Has No Effect on OC-2 Proliferation. Cells were stimulated with various doses of EGF (**A**) or 20 ng/mL of EGF in the presence of various doses of AG1478 (EGFR inhibitor) (**B**), and cell growth was analyzed every 24 h by using WST-1 reagent. Data represent the mean ± SEM (n = 3).
(TIF)

S2 Fig The Effects of Le^y^ Modification on Ligand Binding and Ligand-induced Dimerization EGFR. (**A**) Binding of Alexa-EGF on the cell surface was analyzed using flow cytometry. Unlabeled EGF (2 μg/mL) was used to compete with Alexa-EGF to determine the binding specificity. Mean fluorescence intensity (MFI) of Alexa-EGF binding is shown. Data are presented as the means ± SEM (n = 3). (**B**) Cells were starved and stimulated with EGF (40 ng/mL) for 1.5 min, and the dimerization of EGFR was analyzed. The quantitative data show the ratio of dimer to monomer formation after the indicated durations of EGF (40 ng/mL) treatment. Data are presented as the means ± SEM (n = 3). n.s.: not significant.
(TIF)
