# Supplementary figures and images for: Lewisy Promotes Migration of Oral Cancer Cells by Glycosylation of Epidermal Growth Factor Receptor
Source: PLoS One. 2015 Mar 23;10(3):e0120162. doi: 10.1371/journal.pone.0120162 (PMC4370659; doi:10.1371/journal.pone.0120162)

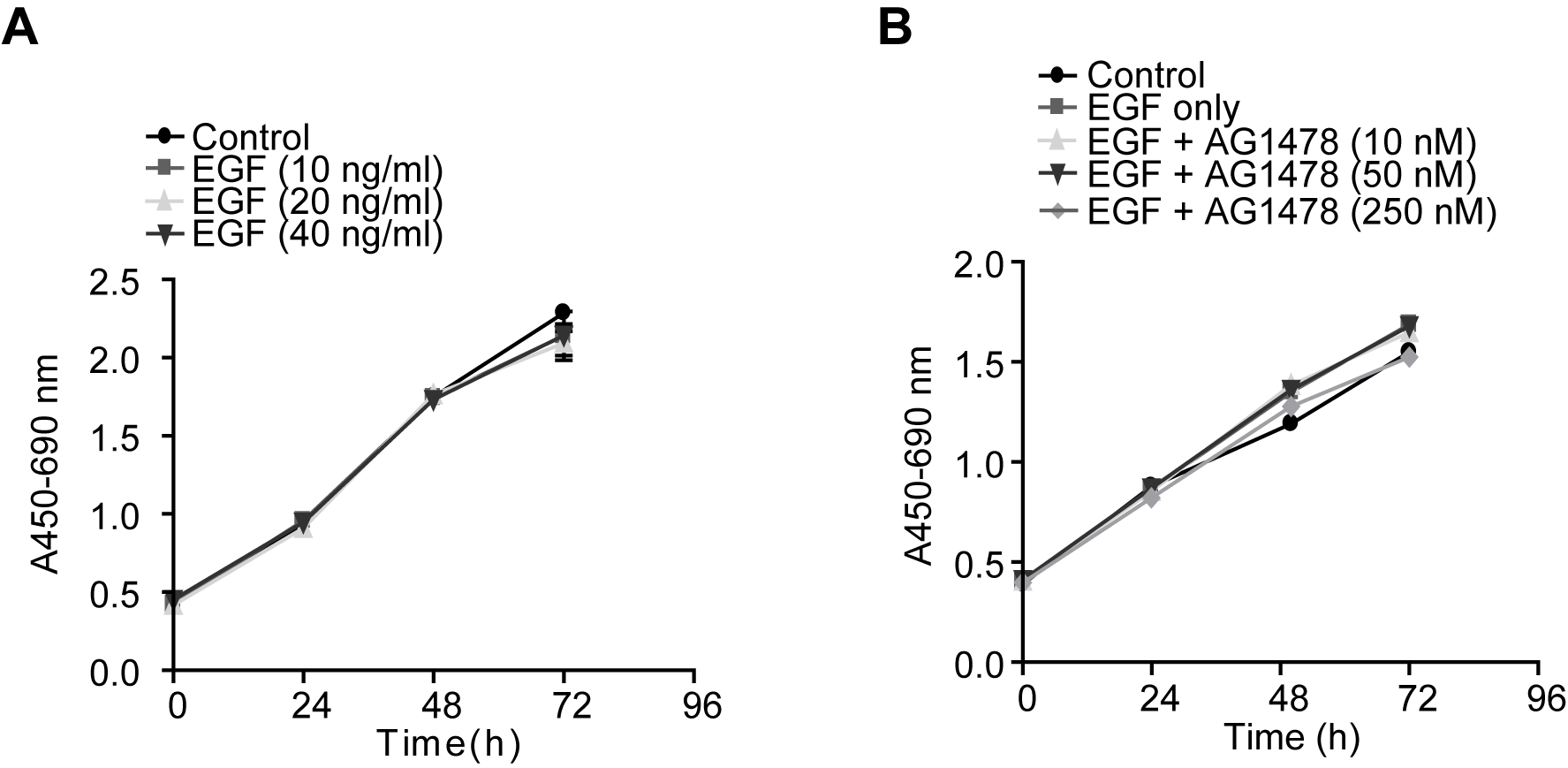

Supplement: S1 Fig — Cells were stimulated with various doses of EGF (A) or 20 ng/mL of EGF in the presence of various doses of AG1478 (EGFR inhibitor) (B), and cell growth was analyzed every 24 h by using WST-1 reagent. Data represent the mean ± SEM (n = 3). (TIF) [file pone.0120162.s001.tif]

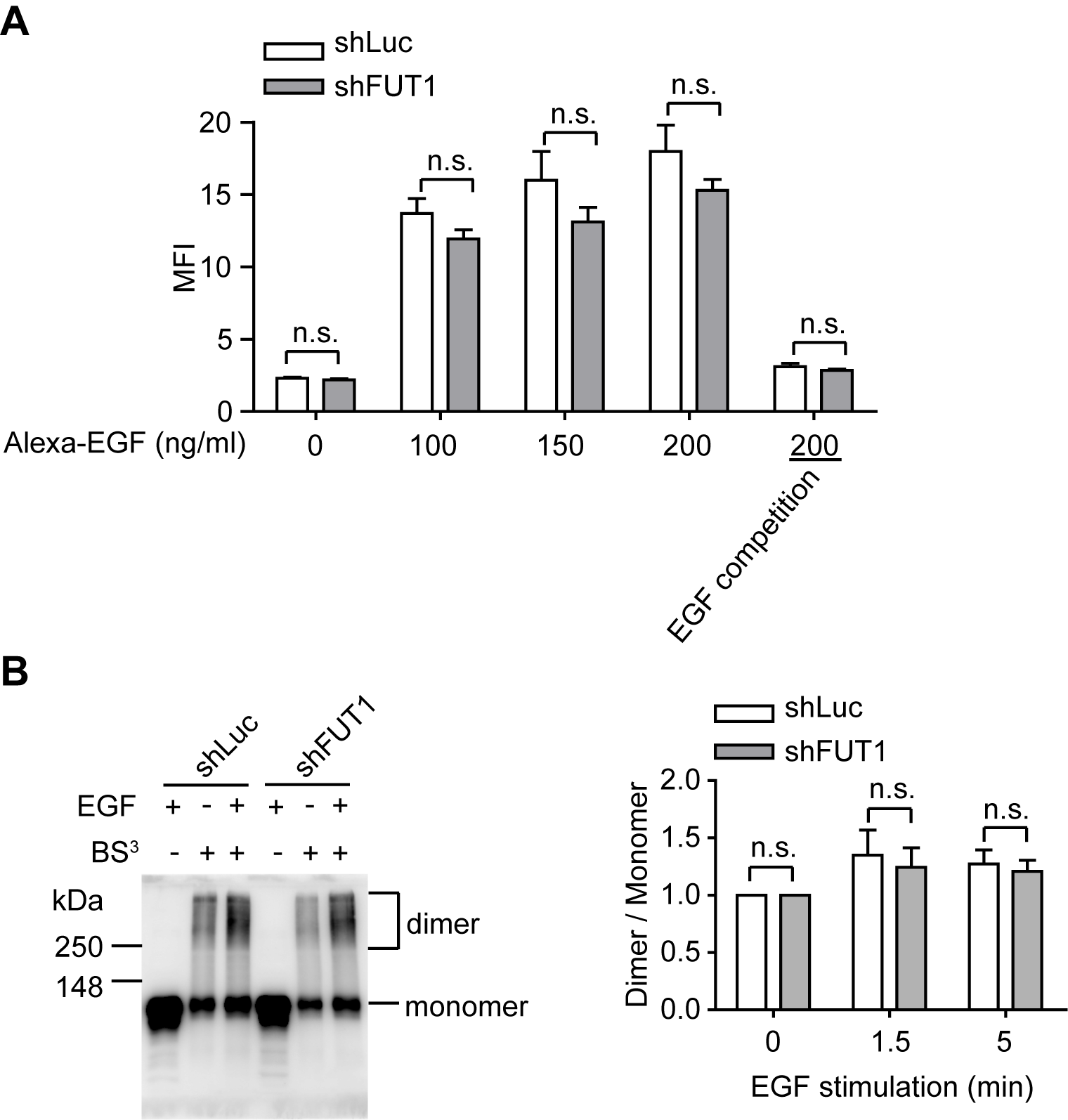

Supplement: S2 Fig — (A) Binding of Alexa-EGF on the cell surface was analyzed using flow cytometry. Unlabeled EGF (2 μg/mL) was used to compete with Alexa-EGF to determine the binding specificity. Mean fluorescence intensity (MFI) of Alexa-EGF binding is shown. Data are presented as the means ± SEM (n = 3). (B) Cells were starved and stimulated with EGF (40 ng/mL) for 1.5 min, and the dimerization of EGFR was analyzed. The quantitative data show the ratio of dimer to monomer formation after the indicated durations of EGF (40 ng/mL) treatment. Data are presented as the means ± SEM (n = 3). n.s.: not significant. (TIF) [file pone.0120162.s002.tif]
